# Targeting of the C-Type Lectin Receptor Langerin Using Bifunctional Mannosylated Antigens

**DOI:** 10.3389/fcell.2020.00556

**Published:** 2020-07-14

**Authors:** Rui-Jun Eveline Li, Tim P. Hogervorst, Silvia Achilli, Sven C. M. Bruijns, Sander Spiekstra, Corinne Vivès, Michel Thépaut, Dmitri V. Filippov, Gijs A. van der Marel, Sandra J. van Vliet, Franck Fieschi, Jeroen D. C. Codée, Yvette van Kooyk

**Affiliations:** ^1^Department of Molecular Cell Biology and Immunology, Cancer Center Amsterdam, Amsterdam Infection and Immunity Institute, Amsterdam University Medical Centers, Vrije Universiteit Amsterdam, Amsterdam, Netherlands; ^2^Department of Bio-organic Synthesis, Faculty of Science, Leiden Institute of Chemistry, Leiden University, Leiden, Netherlands; ^3^Univ. Grenoble Alpes, CEA, CRNS, Institut de Biologie Structurale, Grenoble, France

**Keywords:** mannoside, tumor associated antigens, peptide conjugate, vaccine model, glyco-antigen, Langerhans cell, dendritc cell, langerin

## Abstract

Langerhans cells (LCs) are antigen-presenting cells that reside in the skin. They uniquely express high levels of the C-type lectin receptor Langerin (CD207), which is an attractive target for antigen delivery in immunotherapeutic vaccination strategies against cancer. We here assess a library of 20 synthetic, well-defined mannoside clusters, built up from one, two, and three of six monomannosides, dimannosides, or trimannosides, appended to an oligopeptide backbone, for binding with Langerin using surface plasmon resonance and flow cytometric quantification. It is found that Langerin binding affinity increases with increasing number of mannosides. Hexavalent presentation of the mannosides resulted in binding affinities ranging from 3 to 12 μM. Trivalent presentation of the dimannosides and trimannosides led to Langerin affinity in the same range. The model melanoma gp100 antigenic peptide was subsequently equipped with a hexavalent cluster of the dimannosides and trimannosides as targeting moieties. Surprisingly, although the bifunctional conjugates were taken up in LCs in a Langerin-dependent manner, limited antigen presentation to cytotoxic T cells was observed. These results indicate that targeting glycan moieties on immunotherapeutic vaccines should not only be validated for target binding, but also on the continued effects on biology, such as antigen presentation to both CD8^+^ and CD4^+^ T cells.

## Introduction

Immunotherapeutic vaccination is an appealing approach to direct the immune response toward specific tumor cells. The human skin is an obvious vaccination site for antitumor therapies. Multiple antigen-presenting cell (APC) populations, including Langerhans cells (LCs), CD14^+^ dermal dendritic cells (dDCs), and CD1a^+^ dDCs, are present in the different layers of the skin, where they are key players in the activation of the adaptive immune response. The precise role and the antigen cross-presenting capacity of the different populations remains controversial. In this brief report, we set out to investigate LCs that reside in the epidermis. Langerhans cells, representing 1–5% of the epidermal cells, are considered to be large contributors to *in vivo* antigen cross-priming, compared to other efficient cross-presenting APCs, such as CD141^+^ DCs, which are less prevalent in the skin (Haniffa et al., 2012; Nierkens et al., 2013; Levin et al., 2015). Langerhans cells furthermore play a key role in the induction of T_*H*_1 and T_*H*_17 responses by antigen-specific CD4^+^ T cells (Zaric et al., 2015). Depletion of LCs highly affected therapeutic epicutaneous immunization against cancer cells and reduced the protection by the immune system against tumor growth (Stoitzner et al., 2008).

Langerhans cells specifically express the C-type lectin receptor (CLR) Langerin (CD207) (Santegoets et al., 2008), which is a pattern recognition receptor (PRR), binding carbohydrate structures such as Lewis^*Y*^ antigens and oligomannosides (Feinberg et al., 2011). The Langerin receptor has been targeted for its endocytic and immunomodulatory properties. Liposome functionalization with heparin-derived monosaccharide analogs enhanced Langerin-mediated endocytosis (Wamhoff et al., 2019). By targeted delivery with antibody conjugates, humoral immune responses could be induced, quantified by B-cell activation and antibody isotype switching, as well as promotion of T-follicular helper cell proliferation for B-cell support (Yao et al., 2015; Bouteau et al., 2019). Enhanced antigen presentation to CD8^+^ and CD4^+^ T cells could also be established, which is necessary for T cell-mediated tumor killing in vaccination strategies (Idoyaga et al., 2008). Langerin targeting could also be established with fucosylated synthetic long peptide antigens, which resulted in enhanced antigen presentation by LCs and cross-presentation (Fehres et al., 2017). The exploitation of mannosides for targeting Langerin has been minimally explored due to the presence of many other mannose-binding CLRs, including DC-SIGN (Holla and Skerra, 2011; Varga et al., 2013; Medve et al., 2018).

**FIGURE 1:**
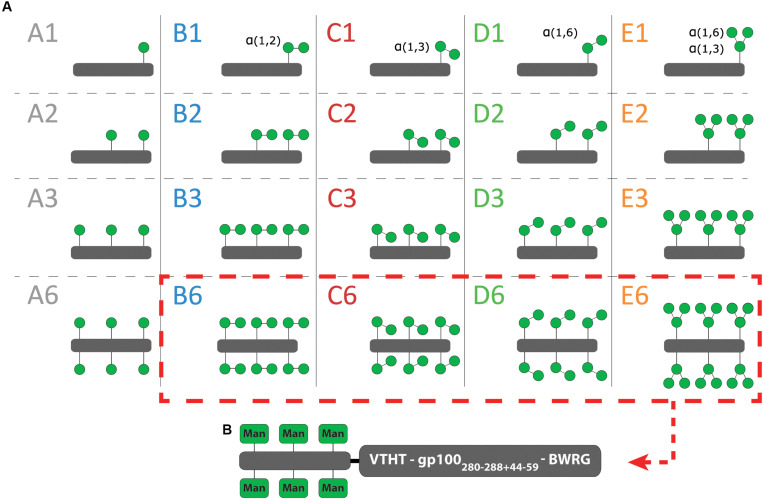
Cartoon of the mannoside clusters. **(A)** A schematic overview of the 20 mannoside clusters. **(B)** Only the hexavalent clusters are further conjugated to a gp100 antigen.

To identify the optimal oligomannoside structure for Langerin targeting, we have used a library of 20 mannose ligands, which we have previously assessed for DC-SIGN binding. The library is built up from clusters of mannosides, appended to a peptide backbone. Five (oligo)mannoside structures (Man; Manα1,2Man; Manα1,3Man; Manα1,6Man; and Manα1,3Man1,6Man saccharides), each representing a substructure of the high affinity Man_9_ oligosaccharide, were used to build the library. The library members systematically vary in saccharide structure (coded A–E) and number of copies on the peptide scaffold (*n* = 1, 2, 3, 6, Figure 1A, and Supplementary Figure 1) (Li et al., 2019).

## Results and Discussion

To determine Langerin extracellular domain (ECD) binding affinity, we measured interactions with surface plasmon resonance (SPR) assays. On the cell surface, Langerin oligomerizes into a trimer for high-affinity ligand engagement (Feinberg et al., 2010). Therefore, in the SPR assays, we made use of a trimeric Langerin ECD attached on the surfaces by the N-termini of their Neck domain, to mimic the natural presentation of the carbohydrate recognition domains at the cell membrane (Feinberg et al., 2011; Porkolab et al., 2020). In the direct interaction mode, where the Langerin ECD was bound to the sensor chip surface facing the ligands in the solvent, the apparent K_*d*_ was calculated for the ligands. Hexavalent presentation of the saccharides corresponded to binding affinities in a 3- to 12-μM range, which is sufficient for Langerin targeting purposes (Table 1). The affinity of the Manα1,6Man glycan D6 was approximately fourfold lower than to the other hexavalent dimannosides (12.3 μM for D6, vs. 3.2 μM for B6 and 3.9 μM for C6), suggesting that Langerin is able to differentiate between the dimannoside structures. This finding is consistent with crystallographic analysis where Manα1,2Man and Manα1,3Man were found binding to the monomeric Langerin CRD, revealing preferential binding of these disaccharides to Langerin (Feinberg et al., 2011). Surprisingly, a minimal affinity difference was observed between the trivalent and hexavalent presentation of the same Manα1,6Man glycan (from 9.8 to 12.3 μM). The same phenomenon was seen with the Manα1,3Manα1,6Man E6 (from 6.5 to 4.23 μM), while the binding affinity of saccharides A and B improved by at least 13-fold from the trivalent to the hexavalent presentation. To quantify binding of the low-affinity ligands, IC_50_ values were assessed with a competition assay using the same trimeric Langerin ECD. Langerin affinity decreased with lower numbers of mannoside copies. Of the monovalent saccharides, only B1 had a binding affinity in the mM range, indicating that the affinity of the other mannosides for Langerin is too weak to be determined in this assay.

**TABLE 1:** Binding affinity to the Langerin receptor.

Ligand	IC_50_ (μM)	Kd_*app*_ (μM)	Ligand	IC_50_ (μM)	Kd_*app*_ (μM)
A1	N.D.		D1	N.D.	
A2	2424 ± 30		D2		16
A3		43	D3		9.8
A6		3.4 ± 1.4	D6		12.3 ± 0.7
B1	4,138 ± 733		E1	N.D.	
B2	181 ± 4		E2		278
B3		48	E3		4.23
B6		3.2 ± 1.7	E6		6.5 ± 2.7
C1	N.D.		N.D. Not Determined		
C2	1,416 ± 13				
C3	281 ± 14				
C6		3.9 ± 1.5			

*Surface plasmon resonance (SPR) analysis demonstrates increase in Langerin affinity with increased multivalency (*n* = 1 > 2 > 3 > 6). All hexavalent clusters bind within an approximate 10 μM range.*

**FIGURE 2:**
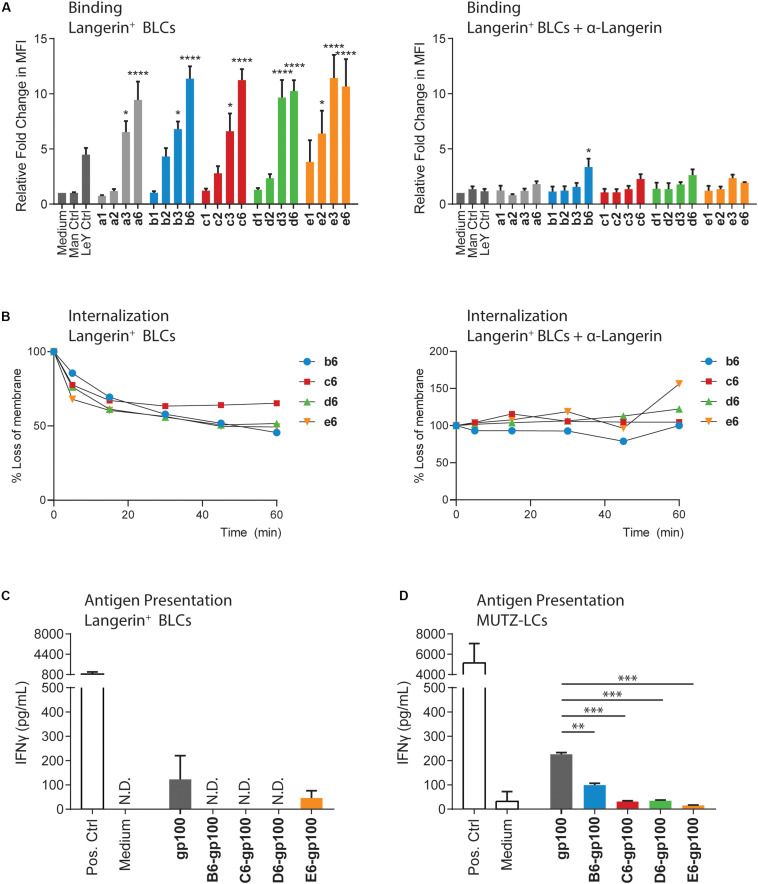
The binding, internalization, and antigen presentation profiles of the mannoside clusters. **(A)** Binding of biotin-functionalized clusters to Langerin^+^ BLCs was measured by flow cytometry. Normalized to medium, the binding profiles mirror the SPR data, where higher multivalent presentation of the saccharides significantly increases the receptor binding compared to medium. The binding is inhibited by blocking with a Langerin-binding antibody. **(B)** Internalization of the biotin-functionalized clusters by Langerin^+^ BLCs was measured by flow cytometry. Internalization was normalized to the unstimulated control. One experiment is shown as representative of three individual experiments. b6, d6, and e6 are internalized after 30 min, whereas c6 remained longer at the surface. Blocking the Langerin receptor prevents the loss from the membrane. **(C)** Antigen presentation by the Langerin^+^ BLCs was measured by IFNγ release of activated T cells. Mannosylated antigens are not presented. Low levels of E6-gp100 activated T cells could be measured, although at lower levels than for the gp100 control. Data are representative of three independent experiments with gp100_280–288_ as positive control and are represented as average ± SD, N.D., not determined. **(D)** Antigen presentation by the MUTZLCs was measured by IFNγ release of activated T cells. Mannosylation of the gp100 antigen significantly reduced antigen presentation compared to the gp100 control. *p < 0.05, **p < 0.01, and ***p < 0.001.

We continued to validate cluster binding on Langerin^+^ cells, using a Langerin-expressing human Epstein–Barr virus-transformed B-lymphoblastic cell (BLC) line (Supplementary Figure 2A; van der Vlist et al., 2011). The cells were pulsed with biotin-functionalized clusters (indicated with lowercase letter codes in Figure 2A) for 30 min at 4°C before staining with fluorescent-labeled streptavidin and flow cytometric quantification. The clusters presenting three or six copies of the Man, Manα1,2Man, and Manα1,3Man saccharides (a3, a6, b3, b6, c3, and c6) showed significantly increased Langerin binding compared to the unstimulated control (Figure 2A), in line with the SPR assay. For the Manα1,6Man and trimannosides, the binding of the trivalent clusters (d3 and e3) was very similar to the binding of the corresponding hexavalent constructs (d6 and e6), as observed in SPR assays. Binding of the clusters could be blocked using a Langerin-specific antibody, confirming Langerin binding specificity of the mannosylated clusters. Taken together, the findings of the binding assays indicate that Langerin affinity depends on the nature of the oligomannosides and that it can be improved through multivalency. Overall, the hexavalent presentation of the mannoside provided ligands with high Langerin affinity. The uptake of these ligands by the Langerin^+^ BLCs was therefore investigated (Figure 2B). The biotin-functionalized clusters were bound for 60 min at 4°C, where after the unbound ligands were washed away with precooled (4°C) medium. The uptake process was initiated by adding warm (37°C) medium to the cells. Cells were sampled on the indicated time points and put on ice to inhibit the uptake processes. The loss of hexavalent clusters on the cell membrane was quantified via flow cytometry after staining with a fluorophore-conjugated streptavidin. Approximately 35–55% of the clusters were internalized after 60 min. Blocking the Langerin receptor or inhibition of receptor-mediated endocytosis by fixing the cells under gentle conditions prevented the loss of the signal (Figure 2B, Supplementary Figure 2C). Targeting of the mannosylated clusters to Langerin thus also triggers Langerin-mediated internalization of the ligands, encouraging the application of these constructs for *in vivo* targeting of the Langerin receptor.

Previously, it has been demonstrated that Lewis^*Y*^-functionalized peptides can be used as vaccine modalities targeting Langerin for enhanced antigen presentation (Fehres et al., 2015). We therefore explored whether the appendage of the mannose clusters to a tumor-associated antigen could have a similar effect. We selected the melanoma-associated gp100 antigen as a well-known tumor-associated model antigen. As previously described, azidolysine-functionalized gp100_280–288+44–59_ antigens were synthesized using automated Fmoc-based solid phase peptide synthesis and conjugated to propargyl-functionalized B6, C6, D6, E6 clusters to generate bifunctional mannosylated antigens (Figure 1B; Li et al., 2019). Four natural occurring amino acids gp100_276–279_ were used as spacer between the two moieties.

Pathogen encounter in the skin causes the LCs to undergo genetic reprogramming. The cell focus shifts from endocytosis to efficient antigen processing and presentation, along with altered cytokine secretion for naive T cell priming and cell migration toward the lymphoid organs (Banchereau et al., 2012; Polak et al., 2014). To study the capacity of the bifunctional conjugates to induce antigen presentation, we quantified T cell activation. A gp100_280–288_ peptide-specific CD8^+^ T cell clone was used in this assay, which secretes interferon γ (IFNγ) upon interaction with the APC-presented gp100 antigen. The BLCs were stimulated for 30 min with the conjugates, where after the compounds were washed away. After overnight stimulation with the T cells, the secreted IFNγ in the medium was measured through enzyme-linked immunosorbent assay (ELISA). The gp100_280–288_ short peptide was used as positive control for antigen recognition by CD8^+^ T cells in major histocompatibility complex (MHC-I) molecules. Surprisingly, minimal T cell activation was measured upon stimulation with the mannosylated antigens, whereas the use of the gp100 peptide as stand-alone antigen was able to induce activation (Figure 2C).

To probe antigen presentation induction by the bifunctional conjugates in a different model, we employed *in vitro*-generated human MUTZ-3 cell line-derived LCs (MUTZ-LCs). The MUTZ-LCs are an established Langerin-expressing model and demonstrate relatively similar binding and internalization kinetics as the Langerin^+^ BLCs (Supplementary Figures 2A,C–E) to study pathogen interactions with, for example, HIV (de Jong et al., 2010; Czubala et al., 2016). After 30-min stimulation with the mannosylated antigens, the MUTZ-LCs were washed and cocultured with the gp100_280–288_-restricted T cell clone, after which T cell activation was measured as described above. In this setup, we were able to measure T cell activation by the bifunctional conjugates (Figure 2D); however, antigen presentation with all mannosylated conjugates was significantly lower than the unglycosylated gp100 antigen, in line with the results obtained in the BLC assay.

These results stand in contrast to previous reports that have demonstrated that Langerin-mediated internalization of Lewis^*Y*^-glycosylated peptide antigens leads to enhanced antigen cross-presentation by human LCs (Fehres et al., 2017; Duinkerken et al., 2019). On the other hand, it has been shown that LCs are not capable of presenting measles virus (MV) and HIV-1 antigens to cytotoxic CD8^+^ T cells (van der Vlist et al., 2011; van den Berg et al., 2015). The glycoproteins decorating the viral envelope of these pathogens are decorated with oligomannosides (Hashiguchi et al., 2007; Bonomelli et al., 2011). Internalization and processing of mannosylated antigens via Langerin may therefore deviate from the uptake and processing of Lewis-antigen conjugates. It is known that, upon Langerin capture, antigens are endocytosed into Birbeck granules, resulting in antigen degradation. Birbeck granules are rod-like structures that are specific to LCs, and they form a component of the endosomal recycling compartment (Thépaut et al., 2009). It has been shown that Langerin-endocytosed HIV antigens are trafficked to Birbeck granules and rapidly degraded (de Witte et al., 2007). This clearance of the virus by Langerin-mediated internalization efficiently prevents HIV-1 and MV transmission to T cells (de Witte et al., 2007; van der Vlist et al., 2011). The mannosylated antigens, under study here, could undergo the same fate, resulting in degradation and minimal antigen presentation and T cell activation as observed here. In contrast, Lewis^*Y*^-functionalized peptides are properly loaded onto MHC-I and cross-presented effectively CD8^+^ T cells (Fehres et al., 2017). Furthermore, whether only one Lewis^*Y*^ moiety was present on the synthetic long peptides, or whether a multivalent Lewis^*Y*^-antigen construct was offered to LCs, enhanced cross-presentation was achieved (Fehres et al., 2017; Duinkerken et al., 2019). These data suggest the involvement of Lewis^*Y*^-mediated routing for successful MHC-I loading and antigen cross-presentation. It thus seems that the nature of the targeting glycan decides the immunological outcome (Geijtenbeek and Gringhuis, 2016).

We have previously described that the mannosylated antigens studied here can be targeted to APCs expressing DC-SIGN, which led to improved antigen uptake. The results obtained with LCs described here indicate that, in an *in vivo* setting, antigen capture by Langerin can contribute to clearance of the conjugate, thereby necessitating the use of a higher vaccine dosage to obtain an adequate cytotoxic CD8^+^ T cell response.

## Conclusion

In summary, we have described the evaluation of a library of oligomannoside clusters for binding to Langerin. In line with previous results, our study has shown an increase in affinity for the lectin with higher multivalent presentation of the mannoside saccharides. Micromolar binding affinity for the hexavalent compounds was measured, as well as Langerin-mediated uptake, demonstrating their applicability as a Langerin-targeting device. Nonetheless, conjugation of the hexavalent mannoside clusters to the gp100 antigen significantly reduced antigen cross-presentation to CD8^+^ T cells in two independent Langerin^+^ APC models, indicating that higher CLR receptor affinity does not have to lead to improved antigen presentation, as we have also observed with DC-SIGN targeting in earlier work (Li et al., 2019). Nevertheless, antigen processing after Langerin-mediated endocytosis and priming of CD4^+^ T cells could provide a different path to boost the immune response. Further work is required to establish whether antigen presentation to CD4^+^ T cells is indeed enhanced, as seen with MV (van der Vlist et al., 2011). Literature implicates the importance of glycan moiety choice for Langerin targeting, as Lewis^*Y*^-functionalized antigens did achieve enhanced Langerin-mediated antigen presentation to cytotoxic T cells (Fehres et al., 2017; Duinkerken et al., 2019). The results presented here emphasize the need to validate the glycan moiety in conjugates not only for receptor binding, but also downstream biological effects, such as antigen presentation to CD8^+^ and CD4^+^ T cells, effectuated by different cell types, before they can be implemented as immunotherapeutic vaccine.

## Materials and Methods

### General Synthesis of the Library

The synthesis of the mannoside library has been described in full in earlier work (Li et al., 2019).

### Cell Culture

Langerin expressing Epstein–Barr virus-transformed BLCs were used as autologous APC (van der Vlist et al., 2011) and were cultured in RPMI 1640 (Invitrogen^TM^) supplemented with 10% fetal calf serum (Biowittaker), 1% penicillin, and streptomycin (both Lonza). Langerin-expressing MUTZ-3-derived LCs were differentiated from the MUTZ-3 progenitor cell line with 100 ng/mL granulocyte-macrophage colony-stimulating factor (Biosource), 10 ng/mL transforming growth factor β (R&D Systems), and 2.5 ng/mL tumor necrosis factor α (Miltenyi) (de Jong et al., 2010).

### Surface Plasmon Resonance Analysis

The ECD of Langerin (residues 68–328) was overexpressed and purified as previously described (Thépaut et al., 2009). The SPR competition experiments were performed on a Biacore T200 using a CM3 series S sensor chip. Control flow cell 1 was functionalized with bovine serum albumin (BSA), whereas flow cell 2 and 3 were treated with 60 μg/mL BSA-Man α1-3[Manα1-6]Man (Dextra) in 10 mM NaOAc pH4 (final densities 2.062 and 2.183 RU, respectively). All flow cells were blocked with ethanolamine. The affinities for the Langerin ECD were evaluated via an inhibition assay, using 25 mM Tris-HCl pH 8, 150 mM NaCl, 4 mM CaCl_2_, and 0.05% P20 surfactant as running buffer. Langerin ECD (20 μM) was injected at 5 μL/min, with or without inhibitor at increasing concentrations. The data were analyzed in Biacore BIAevaluation software using four parameter equation, and the IC_50_ was determined.

To determine the apparent K_*d*_ value, direct interaction experiments were executed on a T200 Biacore with a CM3 series S sensor chip. The Langerin ECD in this assay was functionalized with a StreptagII on the N-terminus (Langerin S-ECD), for oriented capture on the sensor chip surface. The flow cells were functionalized with streptactin protein after EDC/NHS activation. Control flow cell 1 was functionalized with BSA, whereas another flow cell was functionalized with 100 μg/mL Langerin S-ECD via tag-specific capture and simultaneous amine coupling as previously described (Porkolab et al., 2020). An approximate density of about 2.609 RU was achieved on the chip surface. The compounds were injected in increasing concentrations with a flow rate of 30 μL/min in the previously mentioned running buffer. The data were analyzed in Biacore BIAevaluation software for direct interaction 1:1 calculation to determine the apparent K_*d*_ value.

### Mannose Library Binding

Approximately 10^5^ cells were washed and resuspended in 100 μL precooled (4°C) 1 × Hanks balanced salt solution (HBSS; Gibco), supplemented with 1% BSA. The cells were preincubated with 20 μg/mL anti-CD207 [clone 10E2, in-house (de Witte et al., 2007)] in the blocking conditions for 45 min on ice; 10 μM of biotin-functionalized clusters or 1 μg/mL of biotin-functionalized Lewis^*Y*^- or mannose-conjugated polyacrylamide as control was added. After 30-min incubation on ice, the cells were washed with precooled (4°C) phosphate-buffered saline (PBS) and stained with Alexa647-streptavidin (Invitrogen) in PBS supplemented with 0.5% BSA and 0.02% NaN_3_. The cells were extensively washed after 30-min incubation on ice and fixed in PBS supplemented with 0.5% paraformaldehyde (PFA). Fluorescence was measured by flow cytometry (CyAn^TM^ APD with Summit^TM^ software) and analyzed using FlowJo v10.

### Internalization Assay

The cells were harvested and washed with cold (4°C) HBSS, before preincubation with 20 μg/mL anti-CD207 [clone 10E2, in-house (de Witte et al., 2007)] for 45 min on ice in the blocking condition, with 1% PFA in PBS for 20 min on room temperature (RT) in the gently fixated condition, or without pretreatment; 20 μM of the hexavalent biotinylated mannosides was added to the cells in cold HBSS and incubated for 1 h at 4°C. The unbound clusters were washed away with cold HBSS. Warm (37°C) HBSS was added to the cells followed by incubation at 37°C in a shaking heating block. At the indicated time points, a sample was taken and put on ice. The cells were then stained with streptavidin-Alexa647 (Thermo Fisher), and the fluorescence was quantified with flow cytometric analysis (CyAn^TM^ APD with Summit^TM^ software) and analyzed with FlowJo v10.

### Antigen Presentation

Cells were seeded at 50 × 10^3^ cells/well in a 96-well plate (Greiner) and incubated with 20 μM of the peptide conjugates (Supplementary Figure 2F). The gp100 short peptide (gp100_280–288_) was taken along as a positive control. After 30 min of stimulation, cells were washed and cocultured with 10^5^ CD8^+^ HLA-A2.1 restricted T cells from a clone transduced with a gp100_280–288_ specific TCR (Schaft et al., 2003). After overnight stimulation, IFNγ levels in the supernatant were measured by ELISA according to manufacturer’s protocol (Biosource) and measured at 450 nm on the iMark^TM^ Microplate Absorbance Reader (Bio-Rad).

### Statistics

The data are presented as the mean ± SD of at least three independent experiments. Statistical analysis was performed in GraphPad Prism v8. Statistical significance was set at *P* < 0.05 and was evaluated by the Mann–Whitney *U* test.

## Data Availability Statement

The datasets generated for this study are available on request to the corresponding author.

## Author Contributions

R-JL and TH wrote the first drafts of this manuscript. TH synthesized the described constructs under supervision of DF, GM, and JC. SS cultured and monitored the differentiation of the MUTZ-LCs. R-JL determined the cellular affinity, internalization, and antigen presentation aided by SB under supervision of SV and YK. SA and CV performed the SPR experiments under supervision of FF. MT was involved in the preparation of the SPR samples. All authors revised the manuscript.

## Conflict of Interest

The authors declare that the research was conducted in the absence of any commercial or financial relationships that could be construed as a potential conflict of interest.
