# Supplementary material for: Targeting of the C-Type Lectin Receptor Langerin Using Bifunctional Mannosylated Antigens
Source: Front Cell Dev Biol. 2020 Jul 14;8:556. doi: 10.3389/fcell.2020.00556 (PMC7371993; doi:10.3389/fcell.2020.00556)
Supplement: Supplementary file 1 [file Image_1.pdf]

Targeting of the C-Type Lectin Receptor Langerin Using Bifunctional Mannosylated Antigens

Supplementary Material

Supplementary figures 1

Supplementary figures

11 Supplementary Material

Supplementary Figure 1 | Schematic overview of the mannosylated compounds Five mannosides (upper panel) with 1, 2, 3, or 6 copy numbers were used to synthesize the library of 20 constructs (middle panel). The alkyne handle could also be functionalized with a biotin, resulting in a biotynilated cluster (coded with a lower case). In the lower panel, the clusters were functionalized with a gp100$_{280\text{-}288+44\text{-}59}$ antigen.

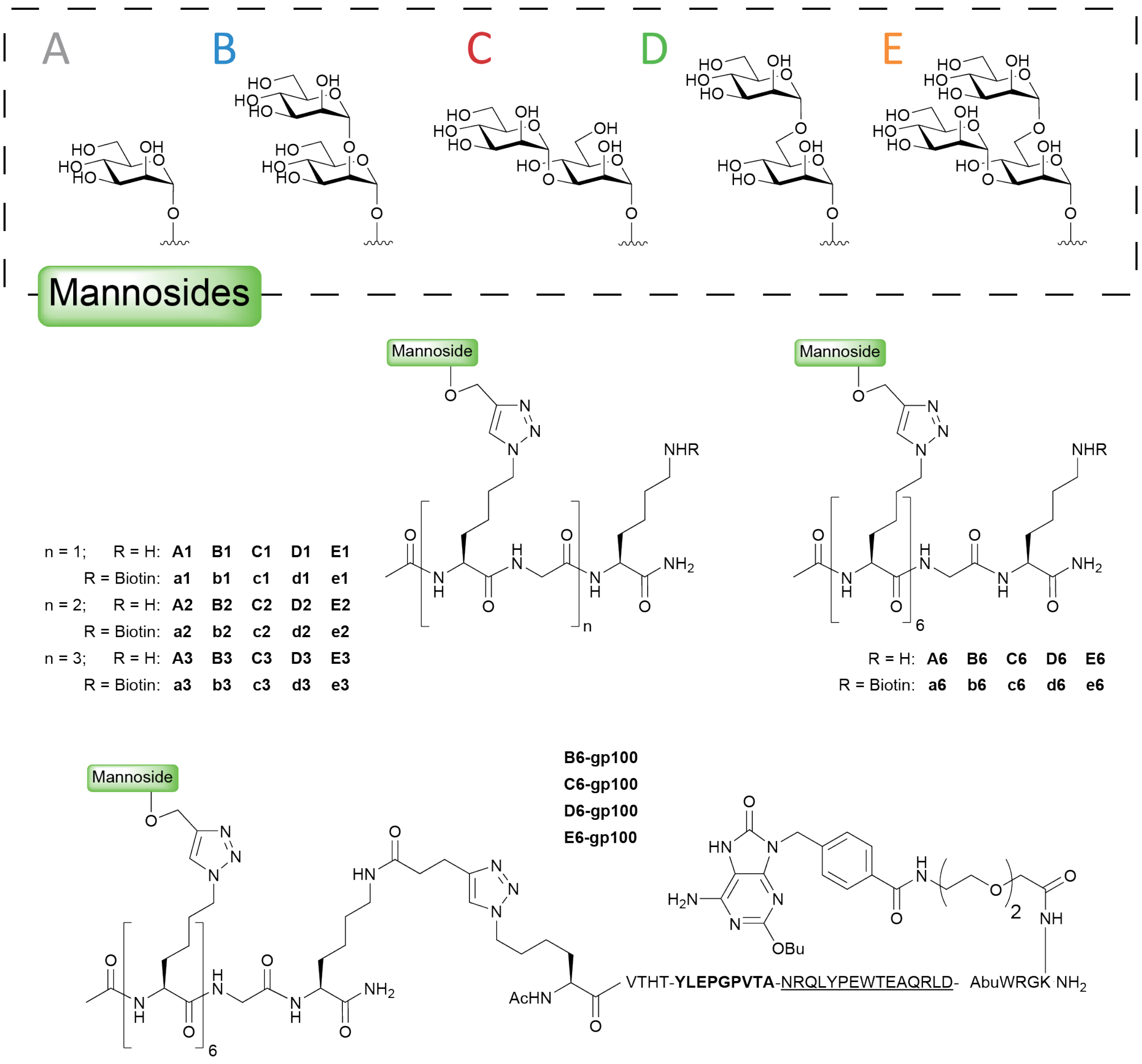

Supplementary Figure 2 | Langerin-, binding-, and internalization profiles of Langerin⁺ BLCs and MUTZ-LCs
(A) Flowcytometric quantification of Langerin and DC-SIGN expression on the BLCs, Langerin transfected BLCs and MUTZ-LCs demonstrate only expression of Langerin in the Langerin⁺ BLCs and MUTZ-LCs, and no expression of DC-SIGN. (B) The internalization of the hexavalent mannoside clusters by gently fixed Langerin⁺ BLCs was measured by flow cytometry, and demonstrates no internalization. (C) Binding of biotin-functionalized clusters to MUTZ-LCs was measured by flow cytometry and normalized to medium (upper panel). The binding is inhibited by blocking with a Langerin blocking antibody (lower panel). (D) Internalization by MUTZ-LCs was measured by flow cytometry, and demonstrate a decrease of 20-45% hexavalent mannoside cluster at the cell membrane. (E) Blocking the Langerin receptor or gently fixing the MUTZ-LCs prevents internalization. One experiments is shown as a representative of 3. (F) The antigenic peptide gp100 was used for antigen presentation with and without a TLR stimulus. 20 µM was chosen because this concentration

provides the largest experimental window.

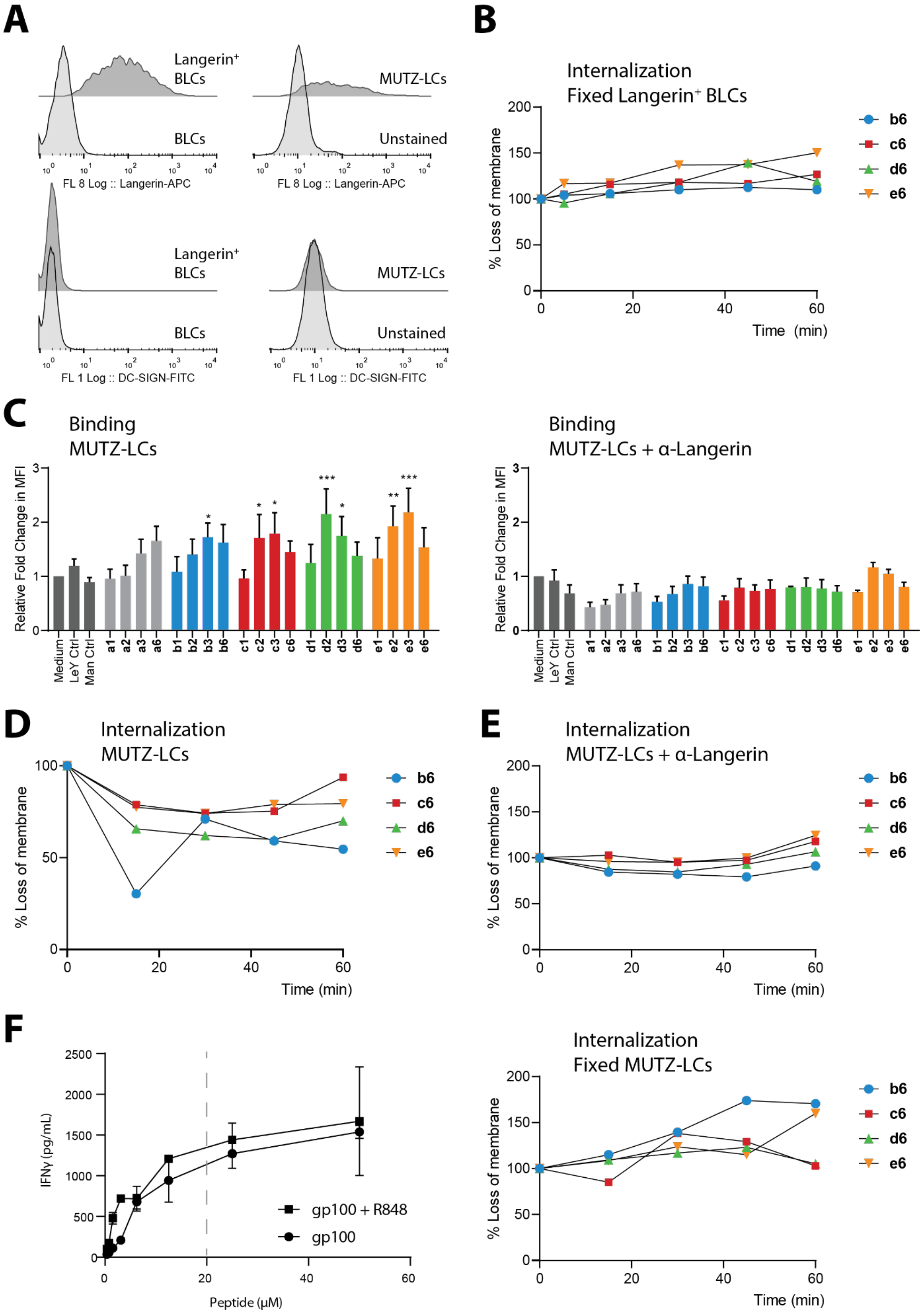

Supplementary Figure 3 | Competition assays (SPR)

It should be taken into consideration that Langerin interacts with the CM-dextran surface and, therefore, no reference subtraction will be done prior to the extraction of binding response.

A1 Langerin

Fc2

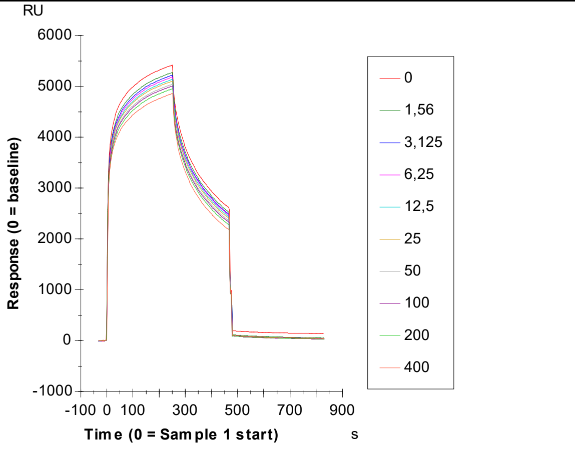

Fc3

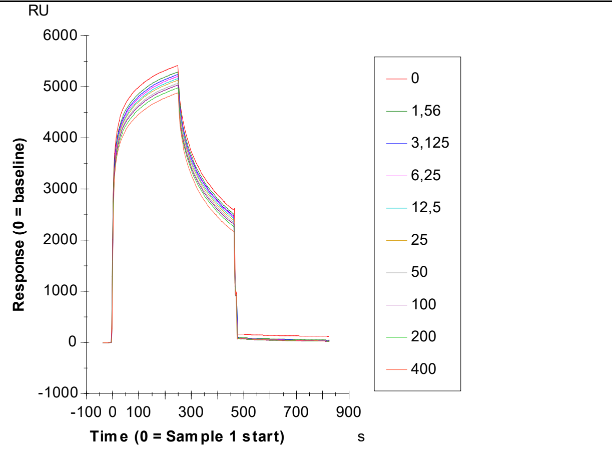

B1 Langerin

Fc2

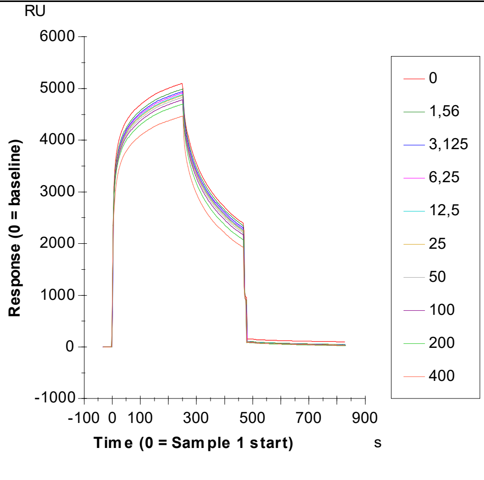

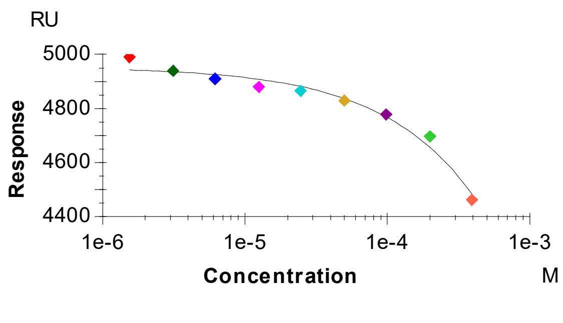

Fc3

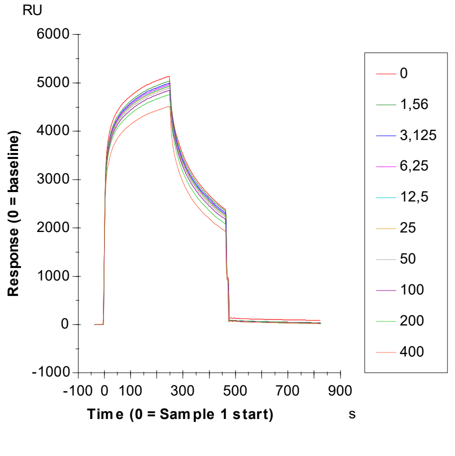

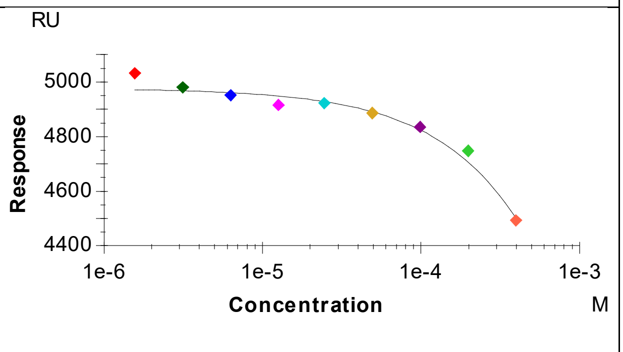

C1 Langerin

Fc2

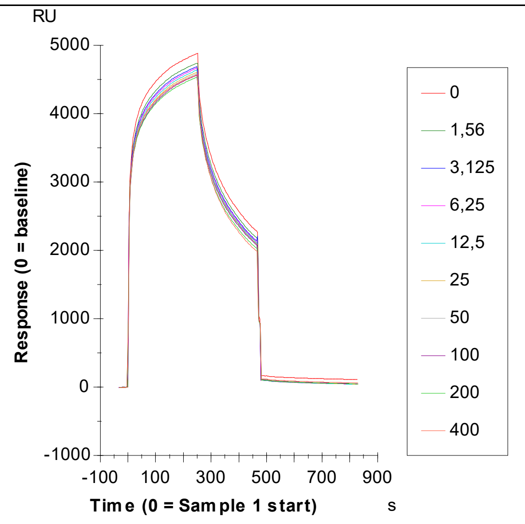

Fc3

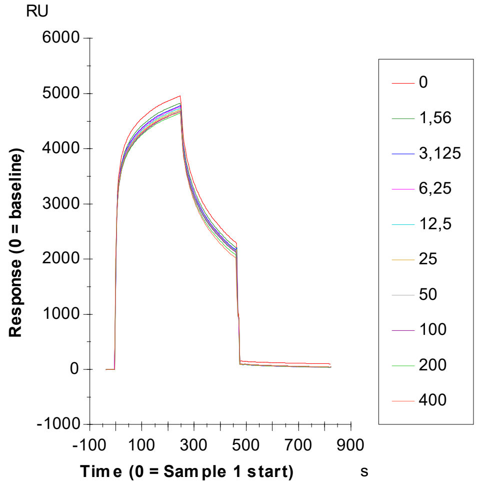

D1 Langerin

Fc2

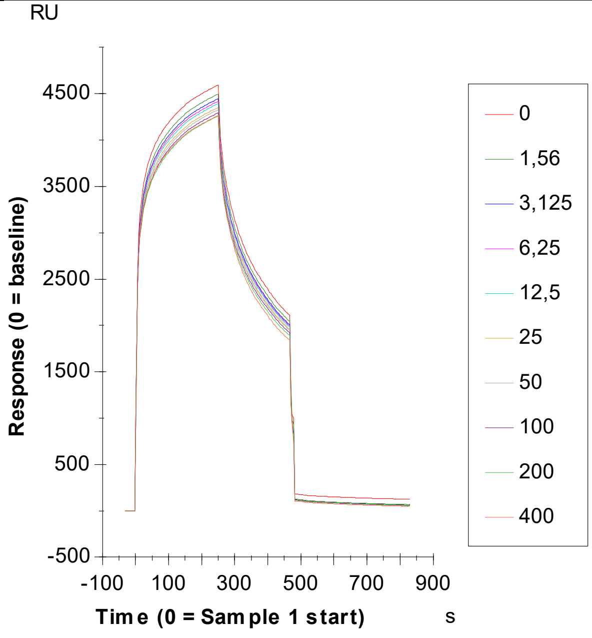

Fc3

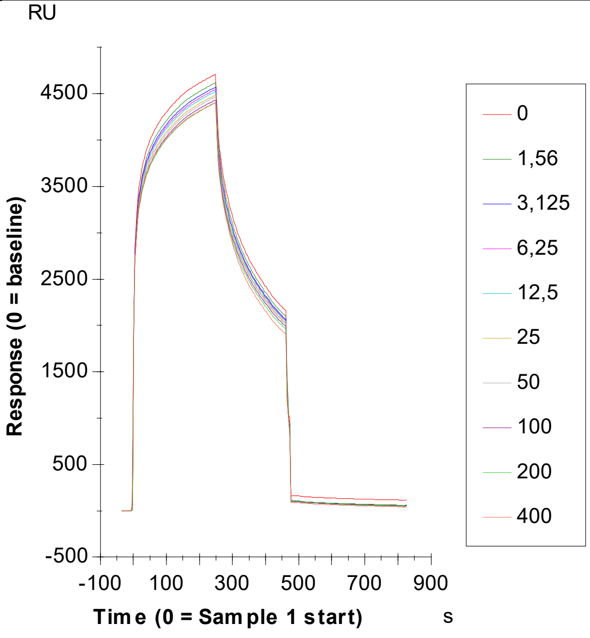

E1 Langerin

Fc2	Fc3
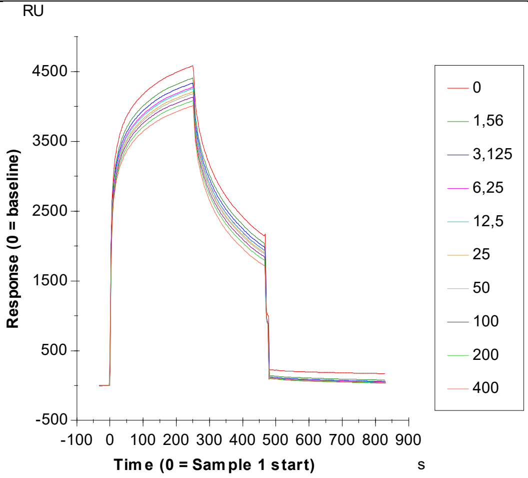	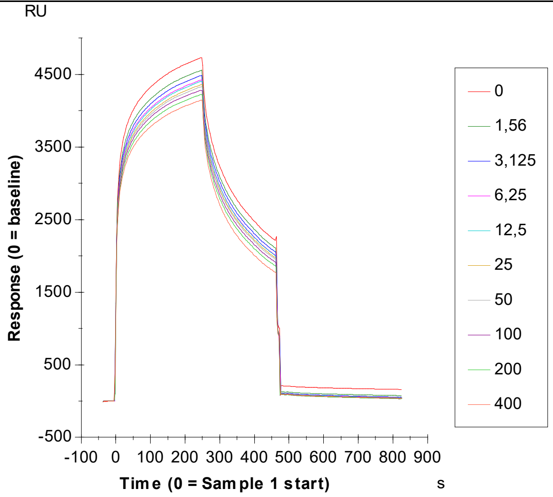

A2 Langerin

Fc2	Fc3
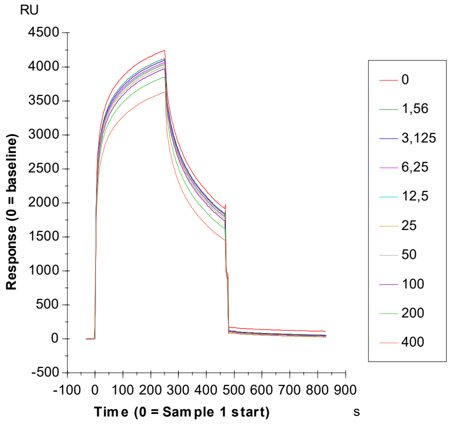	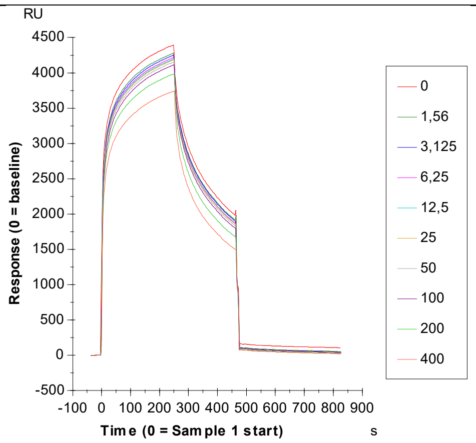
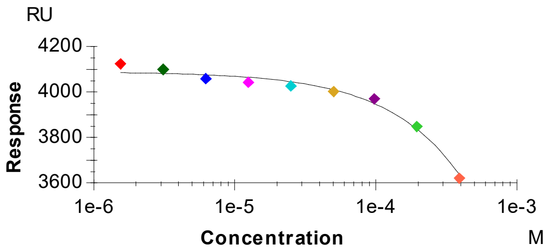	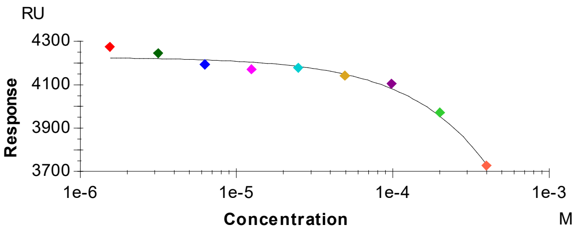

B2 Langerin

Fc2

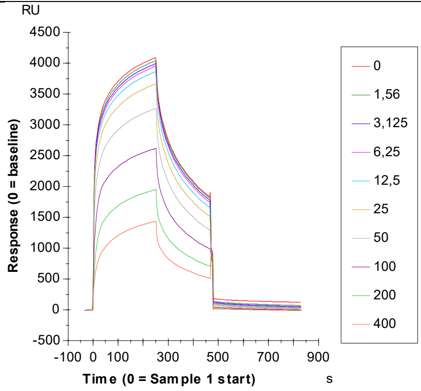

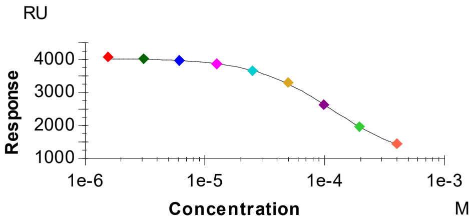

Fc3

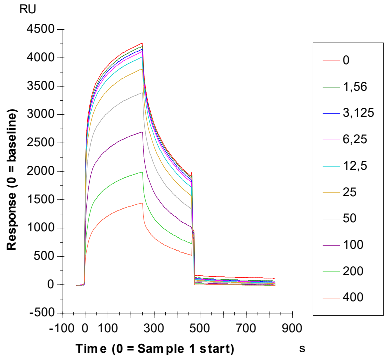

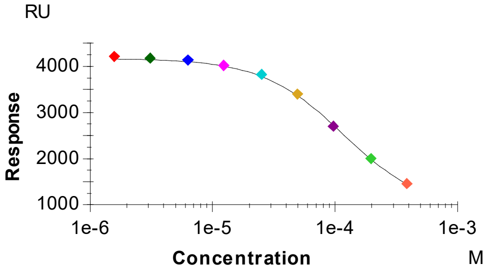

C2 Langerin

Fc2

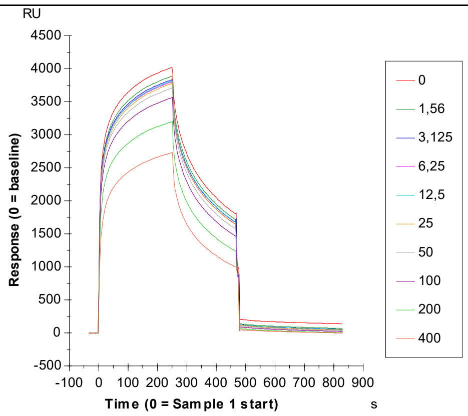

Fc3

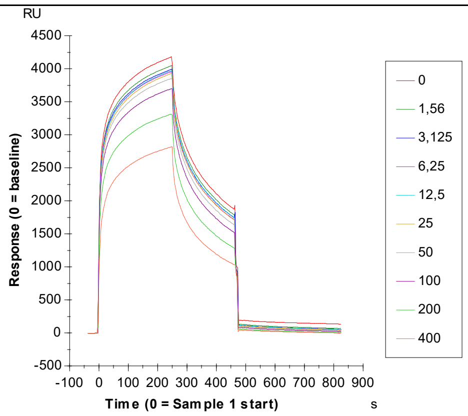

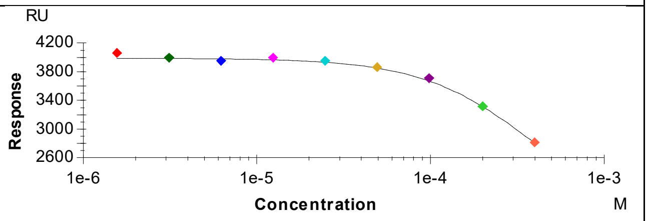

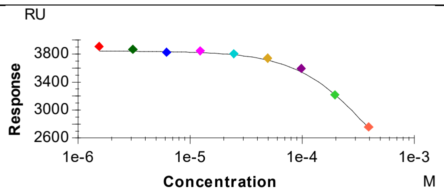

C3 Langerin

Fc2	Fc3

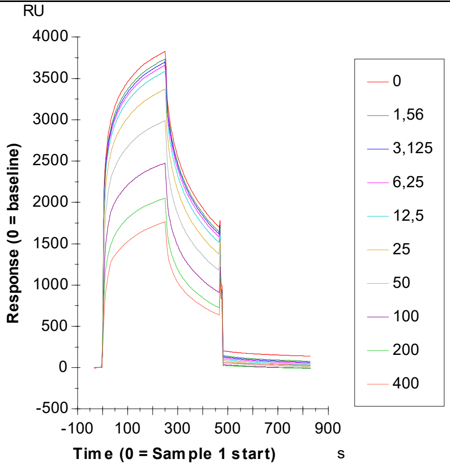

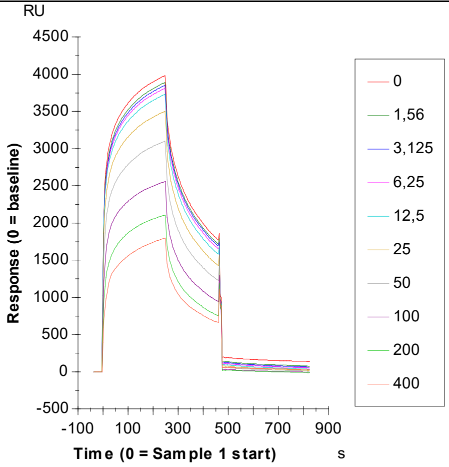

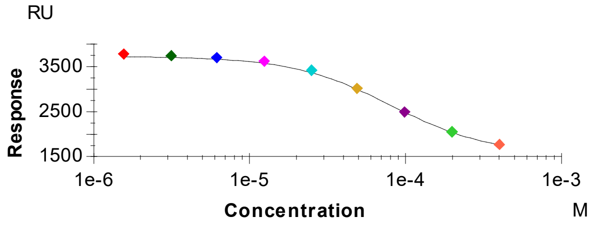

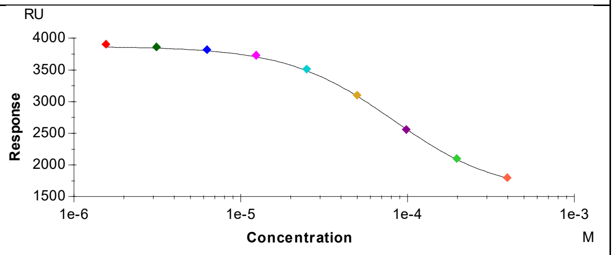

Supplementary Figure 4 | Direct interaction assays (SPR)

A6

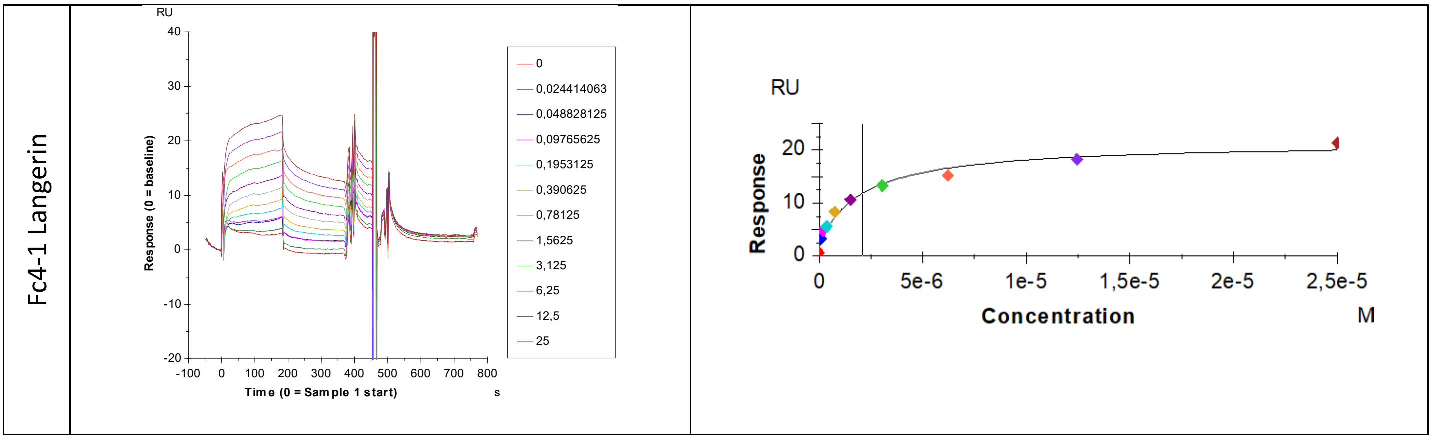

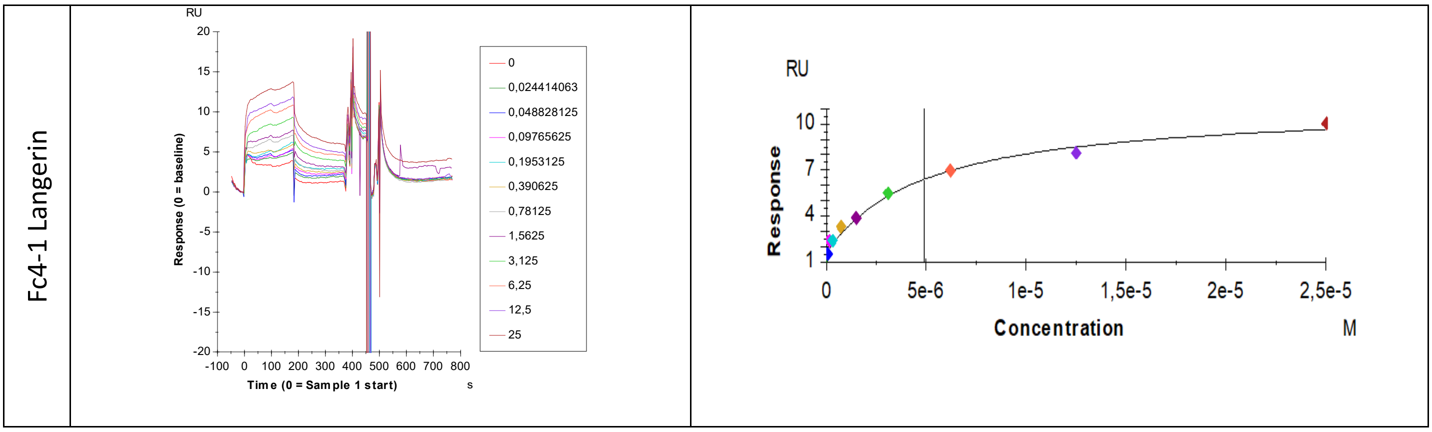

B6

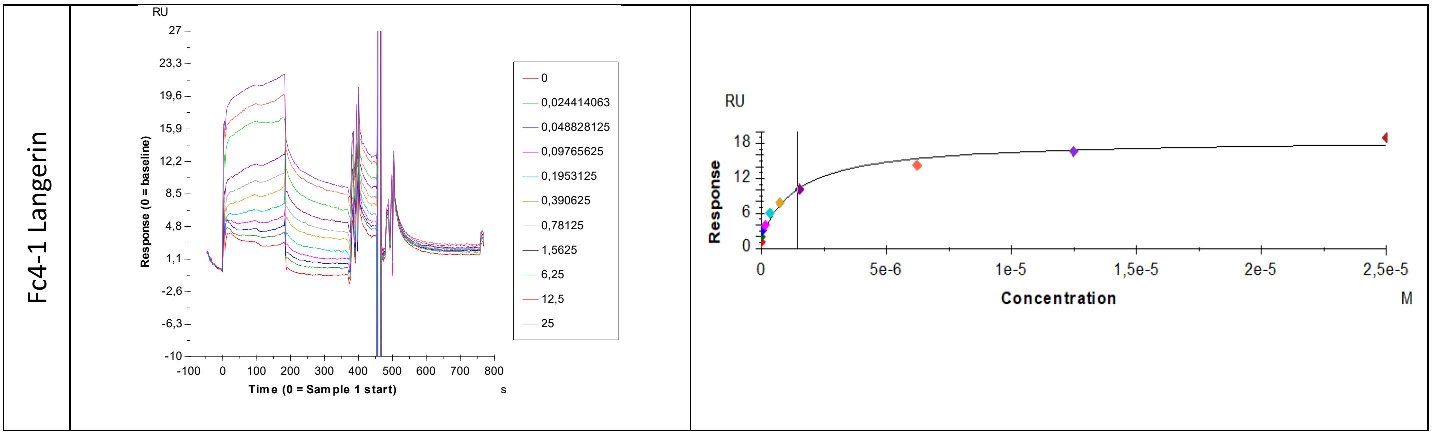

Fc4-1 Langerin

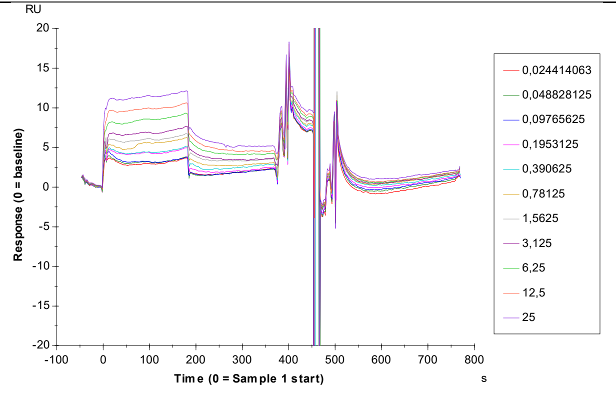

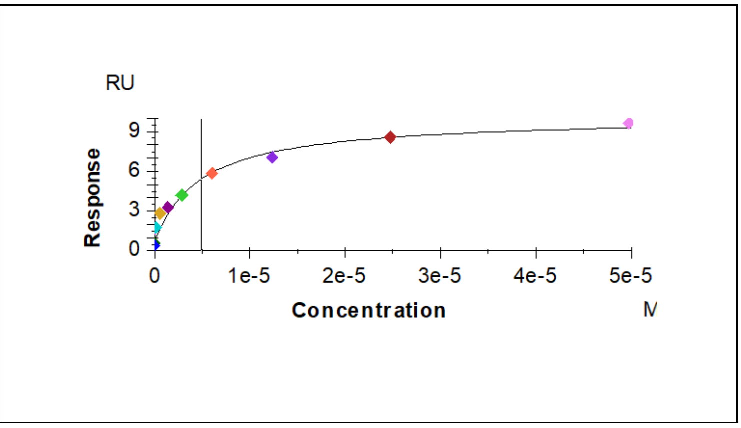

C6

Fc4-1 Langerin

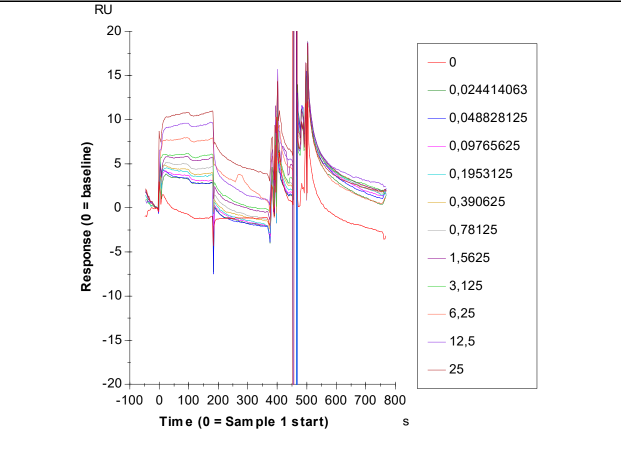

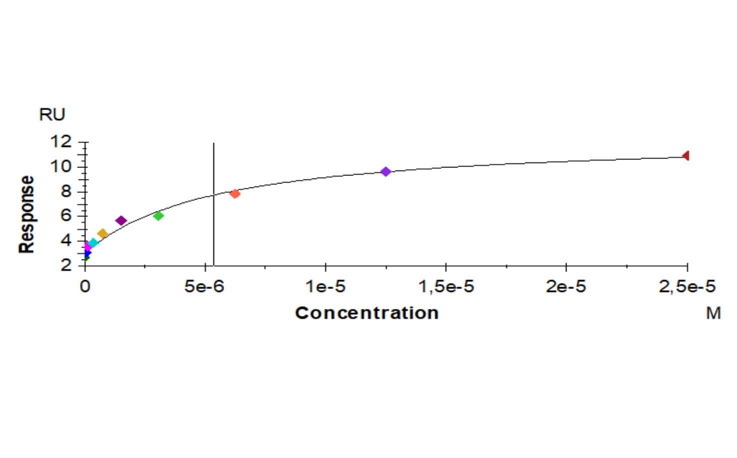

Fc4-1 Langerin

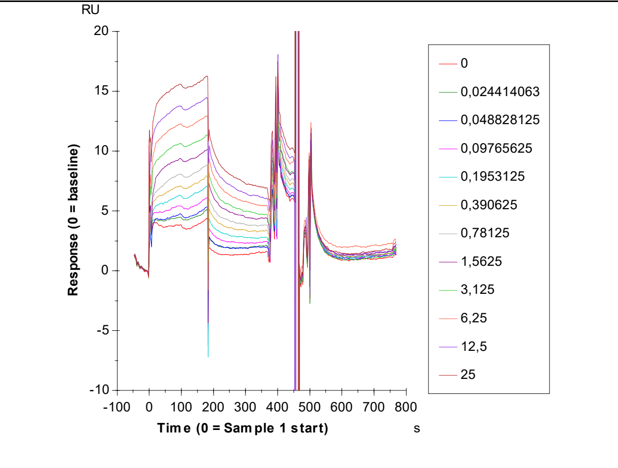

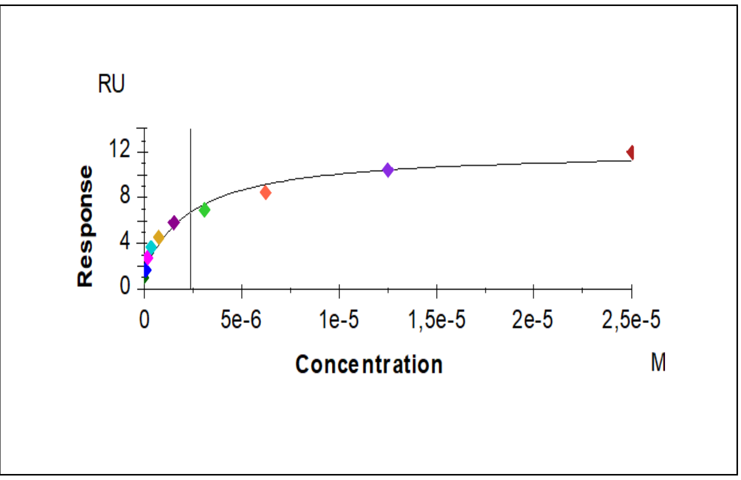

D6

Fc4-1 Langerin

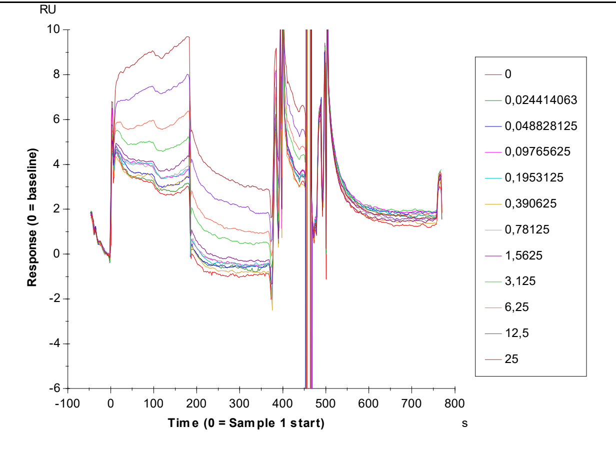

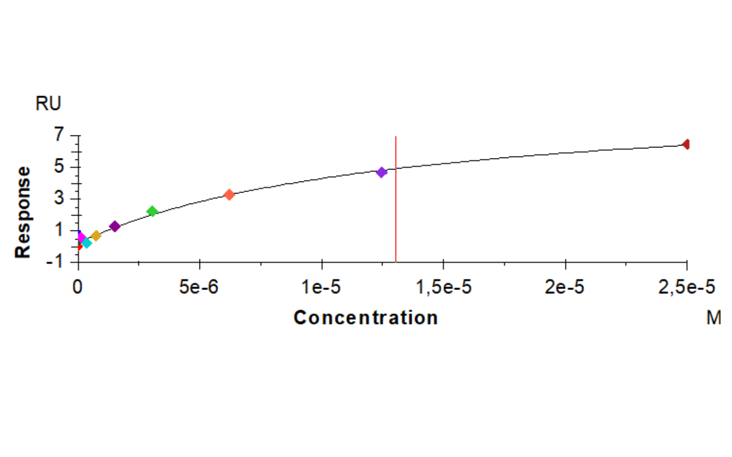

Fc4-1 Langerin

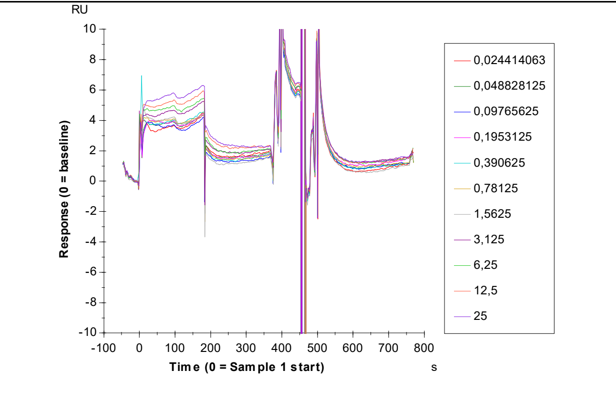

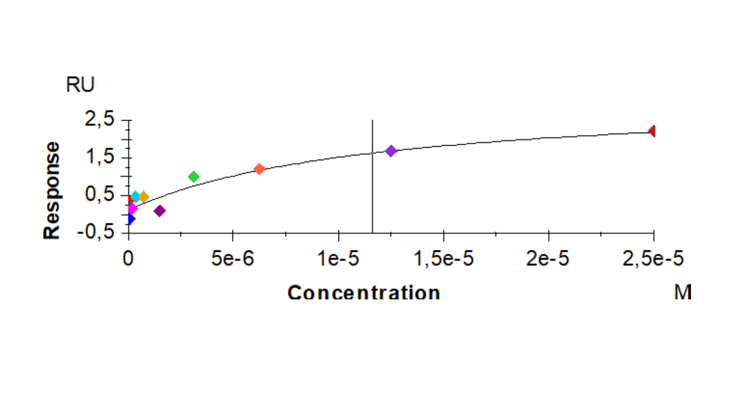

E6

Fc4-1 Langerin

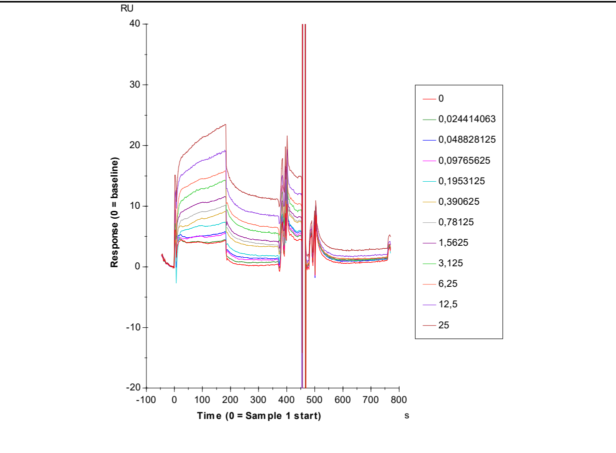

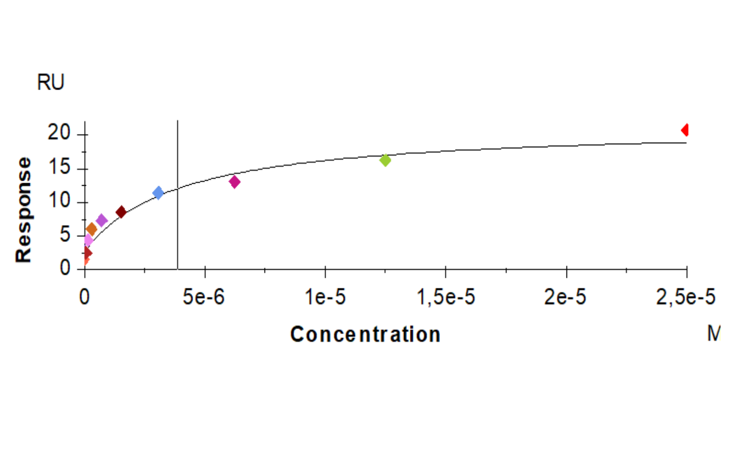

Fc4-1 Langerin

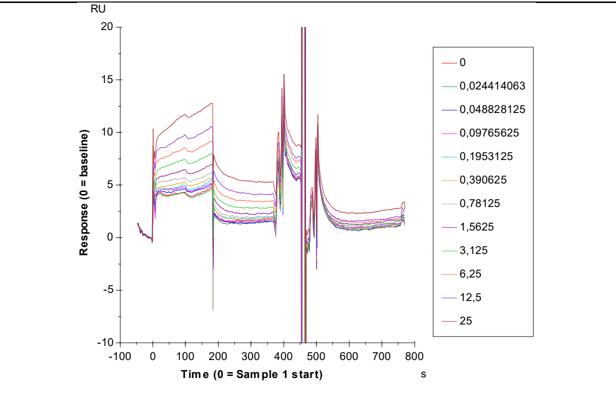

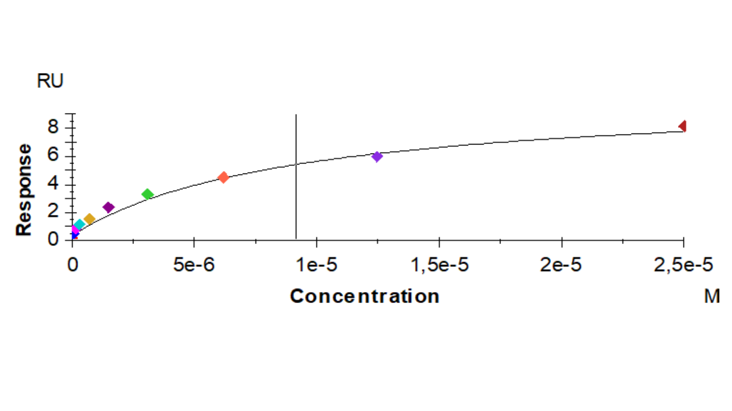

F6

Fc4-1 Langerin

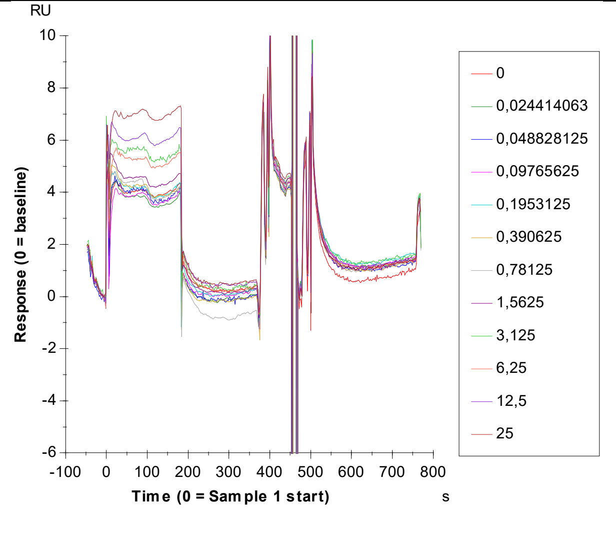

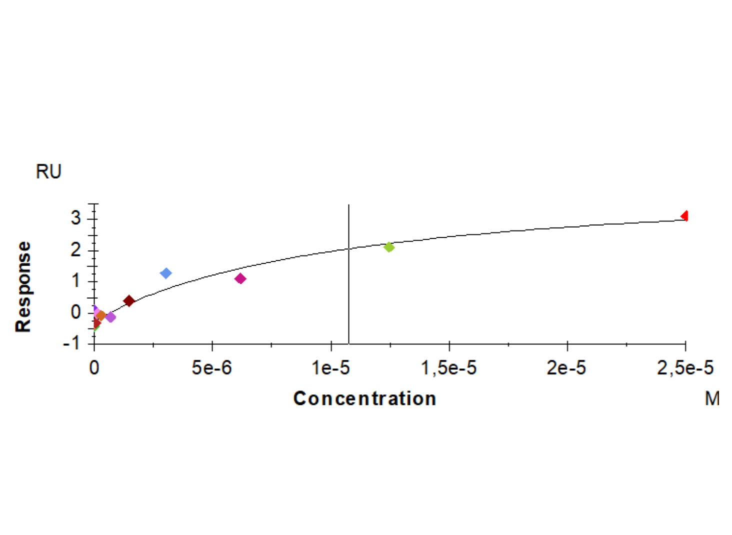

A3

Fc4-1 Langerin

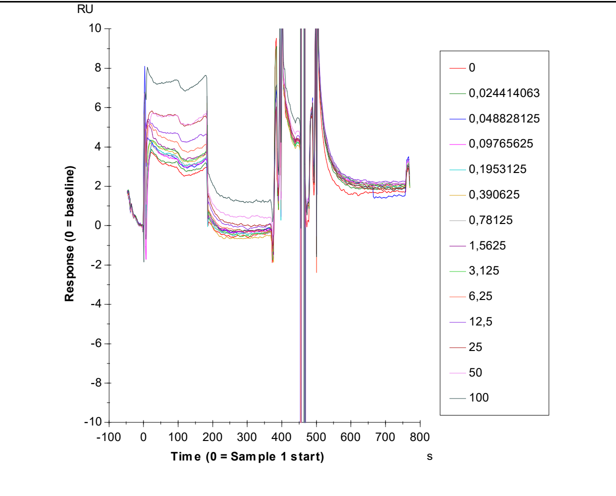

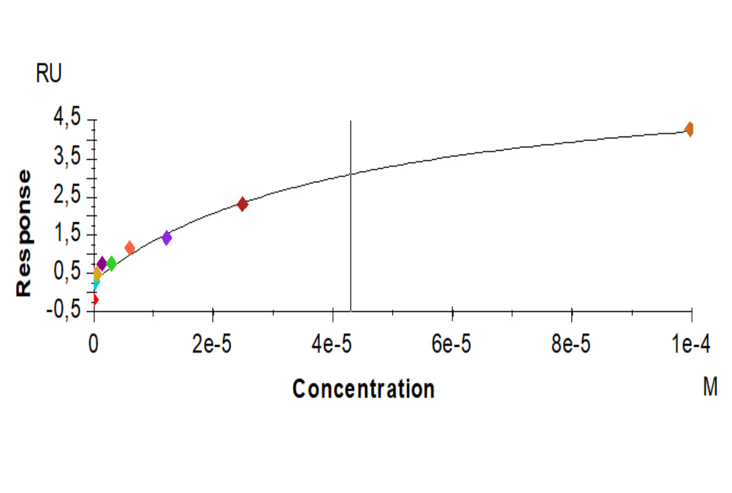

B3

Fc4-1 Langerin

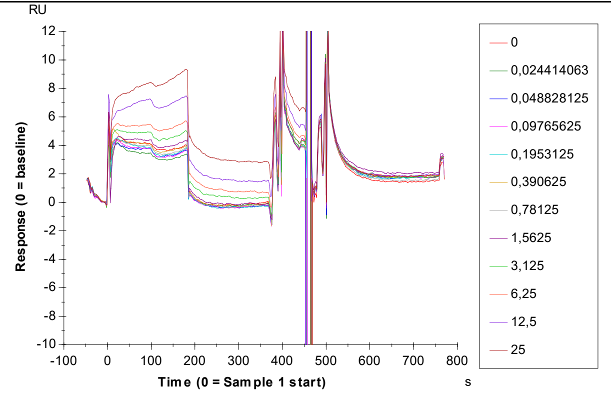

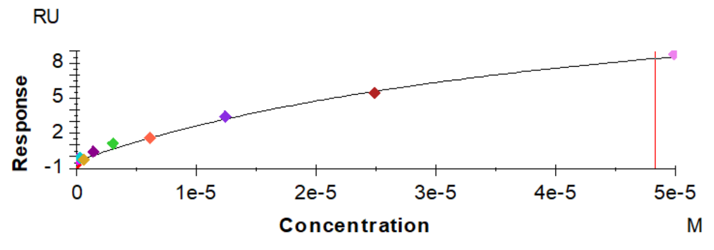

C3

Fc4-1 Langerin

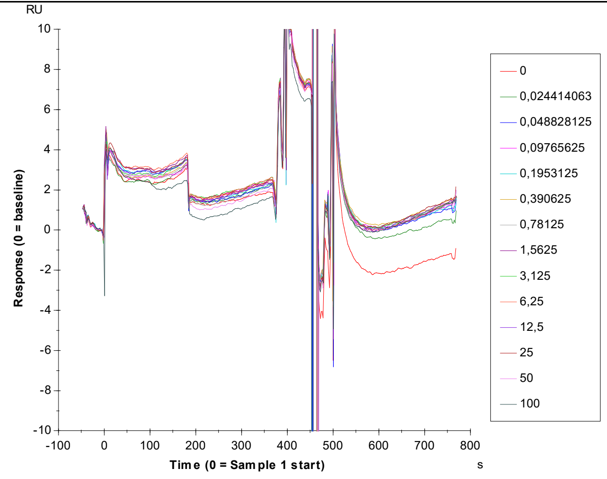

Not possible to determine

D3

Fc4-1 Langerin

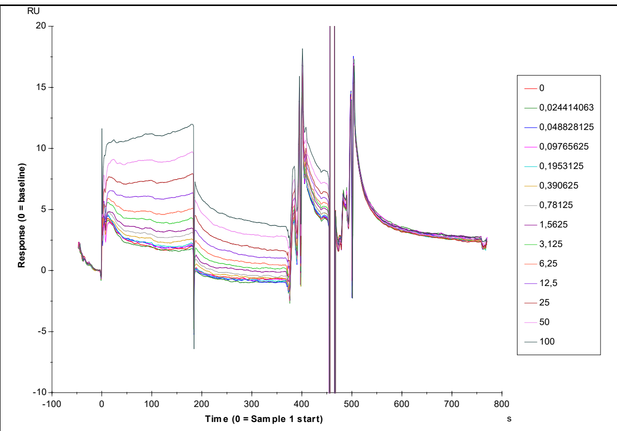

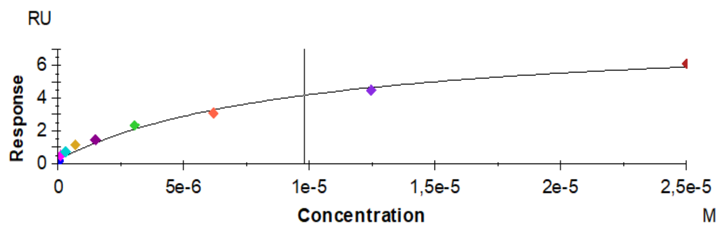

E3

Fc4-1 Langerin

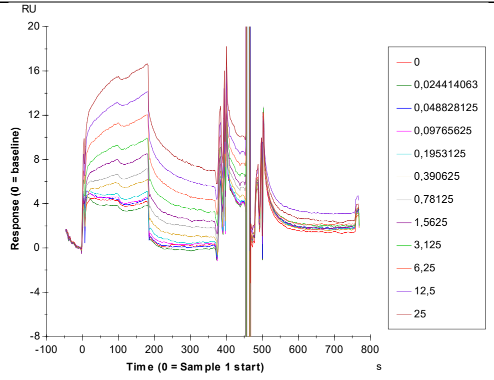

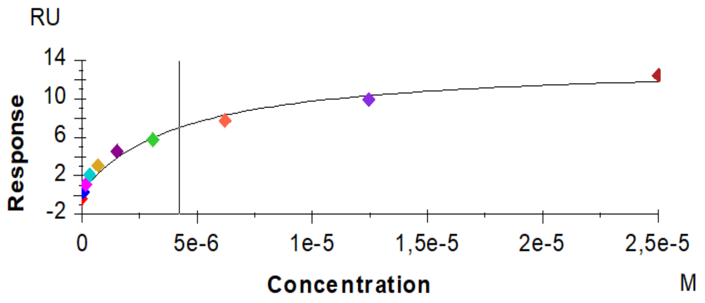

B2

Fc4-1 Langerin

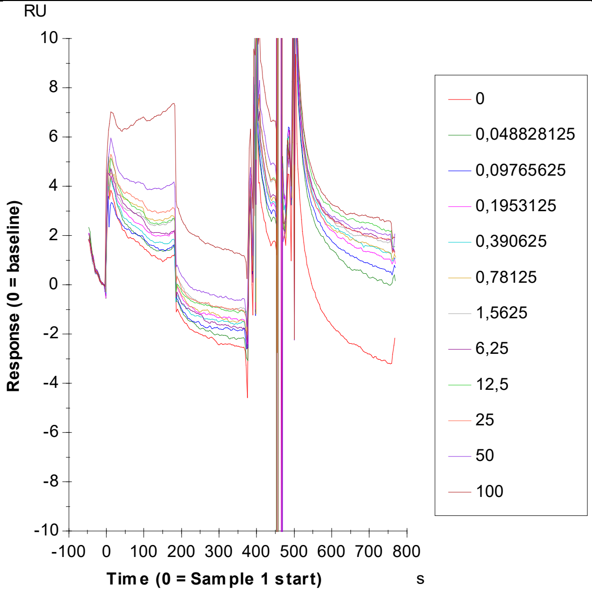

Not possible to determine

C2

Fc4-1 Langerin	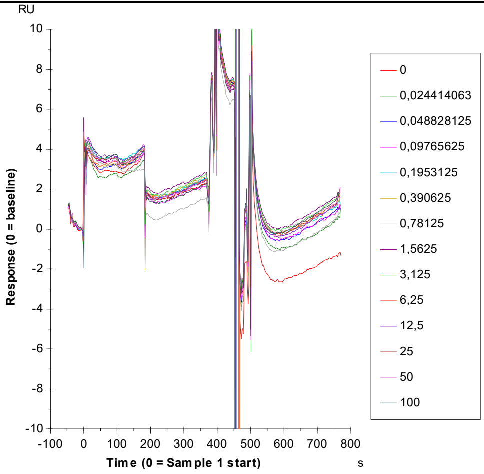	*Not possible to determine*

D2

Fc4-1 Langerin	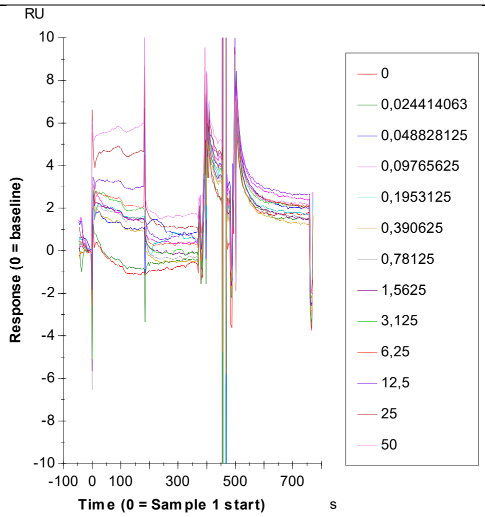	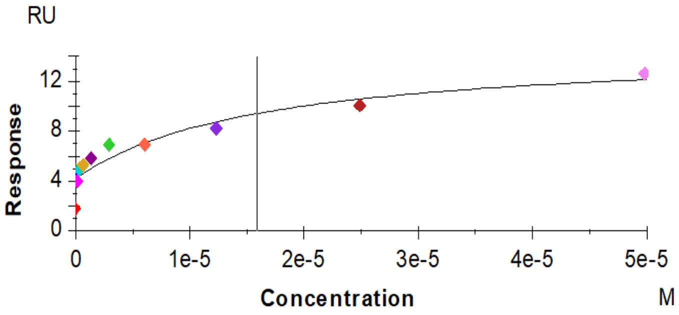

E2

Fc4-1 Langerin

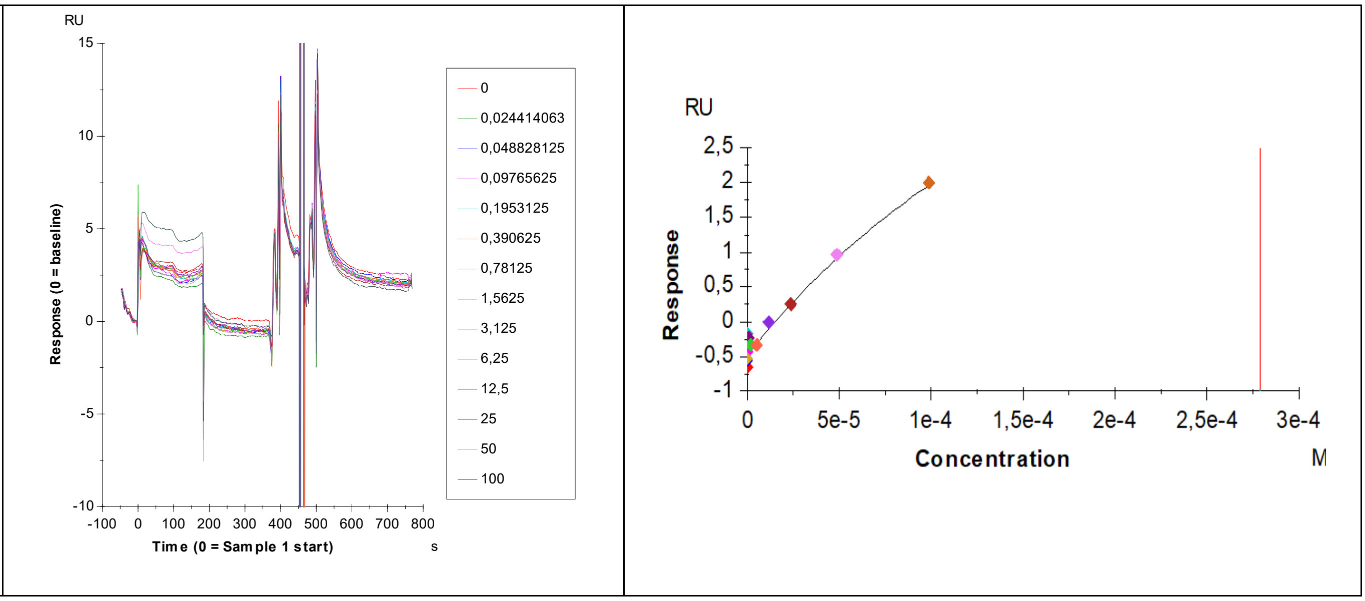

B1

Fc4-1 Langerin

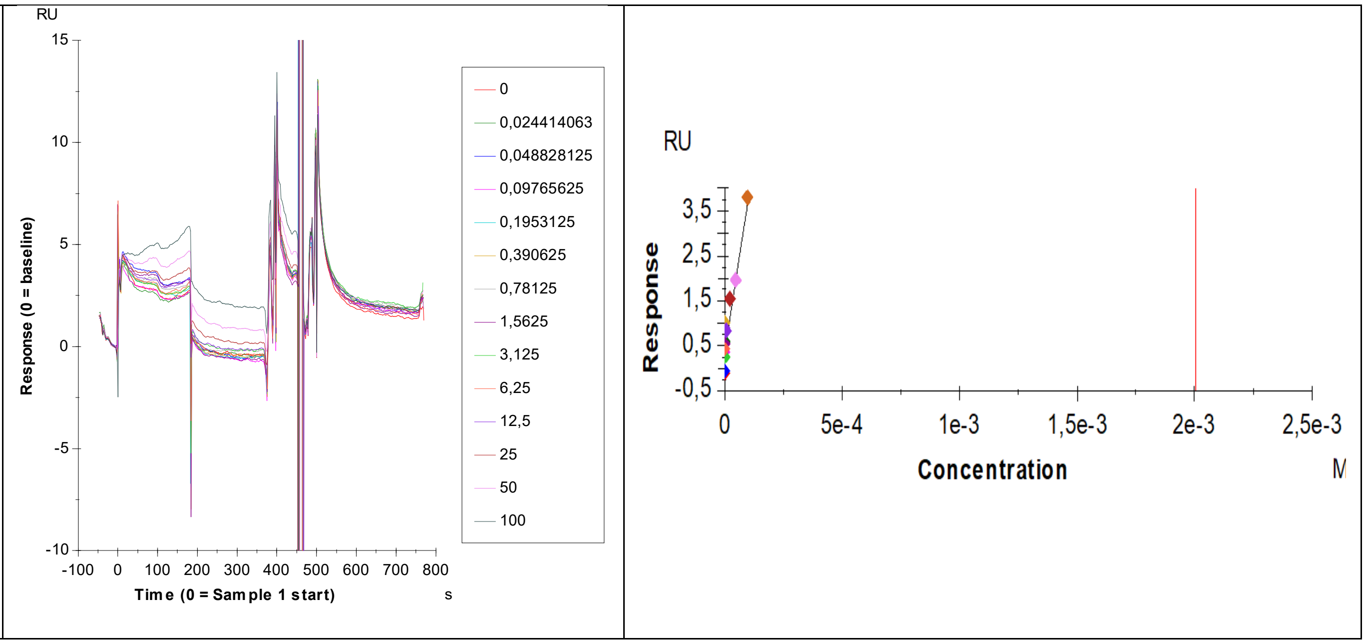

E1

Fc4-1 Langerin	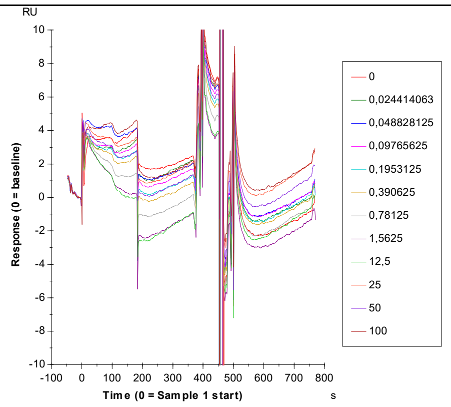	*Not possible to determine*

D1

Fc4-1 Langerin	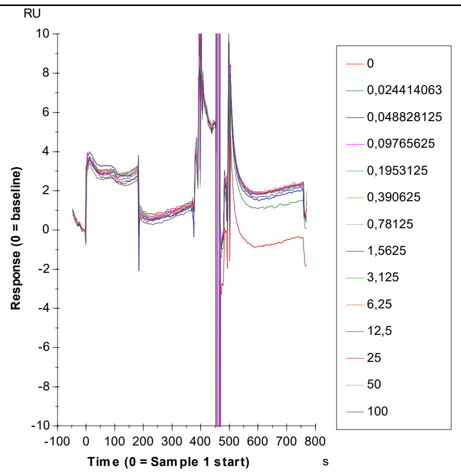	*Not possible to determine*
